# Developing Glow: A Co‐Designed Support Framework Integrating Behavioural and Psychological Needs in Coeliac Disease

**DOI:** 10.1111/hex.70905

**Published:** 2026-10-08

**Authors:** Rose‐Marie Satherley, Shakira Hollyfield, Areeba Shahab, Angie Pitt, Nick Trott, Lizz Toft, Aftab Ala, Christina J. Jones

**Affiliations:** ^1^ School of Psychology, Department of Psychological Interventions Faculty of Health and Medical Sciences, University of Surrey Guildford UK; ^2^ Department of Psychological Medicine Institute of Psychiatry, Psychology & Neuroscience, King's College London London UK; ^3^ Academic Unit of Gastroenterology, Sheffield Teaching Hospitals Sheffield UK; ^4^ Department of Nutrition & Dietetics Royal Surrey NHS Foundation Trust Guildford UK; ^5^ Institute of Liver Studies, King's College Hospital London UK

**Keywords:** behavioural, coeliac disease, dietetic, psychological

## Abstract

**Background:**

Strict management of a gluten‐free diet (GFD) is the only effective treatment for coeliac disease; support in current healthcare frameworks focususes primarily on dietary support and intestinal recovery. However, psychological and social processes that shape gluten‐free living are less often addressed, despite high levels of emotional burden among people with coeliac disease. There is limited guidance on how these needs should be met in routine support.

**Objective:**

To co‐design an evidence‐informed framework integrating dietary, behavioural and psychological support for adults living with coeliac disease.

**Design:**

Co‐design, using iterative workshops and interviews, underpinned by a constructivist epistemology and a reflexive framework approach. The Theory of Planned Behaviour and Acceptance and Commitment Therapy were used as sensitising lenses to interpret behavioural and experiential mechanisms.

**Setting and Participants:**

Sixty‐two stakeholders in the UK, including 35 adults with coeliac disease and 27 healthcare providers. Recruitment prioritised groups under‐represented in coeliac research.

**Results:**

Analysis produced three interpretive themes: (1) fragmented and inconsistent support landscapes shape expectations, confidence, and responsibility; (2) gluten‐free living requires ongoing identity reconstruction amid misrecognition and life transitions; and (3) GFD management involves sustained cognitive, emotional and relational work, with fluctuating capacity. These informed development of the GLOW (Gluten‐free Living: Optimised Wellbeing) framework, which defines three dimensions of support: Orientation and Adjustment, Sustained Support, and Specialist Psychological Input. GLOW describes support functions rather than roles, to enable delivery across healthcare, community, digital, and voluntary‐sector settings.

**Conclusions:**

For adults with coeliac disease, GFD management involves ongoing psychological work that is insufficiently addressed by current models of care. GLOW offers a mechanism‐oriented approach to integrate behavioural and emotional support into routine coeliac disease services and wider support environments. Future work will evaluate implementation and resource requirements across settings.

**Patient or Public Contribution:**

People with lived experience of coeliac disease were involved throughout the study, including design, data collection, analysis, and development of the GLOW framework. Two lived experience advisors contributed as members of the Project Advisory Panel, providing input into study design, interpretation of findings, and refinement of the framework, and another as a lived experience researcher. Adults with coeliac disease also participated in co‐design workshops and interviews, shaping the identification of key challenges, support needs, and proposed solutions. Wider feedback on the framework was obtained through engagement with community groups and Coeliac UK networks.

## 1 Introduction

Coeliac disease is an autoimmune condition affecting at least 1 in 100 people worldwide [1]. For those with coeliac disease, eating gluten causes damage to the small intestine. Untreated, the condition is associated with nutritional deficiencies, osteoporosis and fracture risk, malignancy, reproductive complications, neurological manifestations, and increased risk of other autoimmune conditions [1]. The only treatment is lifelong self‐management of a gluten‐free diet (GFD). However, most people with coeliac disease will experience intermittent gluten exposure [2]. Gluten is present in wheat, barley, rye and most oats, making avoidance challenging given the widespread use. To minimise risk, individuals must consistently monitor food ingredients, food preparation, and self‐advocate in social situations [2]. Self‐management is further complicated by inconsistent food‐labelling laws [3], poor awareness of the GFD in food‐service industry [4], and the widespread misconception that a GFD is a lifestyle choice rather than a treatment [5, 6].

As well as these practical challenges, psychological comorbidity is increasingly documented. A meta‐analysis reported that adults with coeliac disease had more than twice the risk of depression and up to six times the risk of anxiety compared with the general population [7]. Symptoms of depression can persist for some, and are associated with reduced motivation and capacity to maintain a GFD [8]. For others, concerns about gluten cross‐contact can drive avoidant eating that limits gluten exposure but is also associated with social restriction and distress [9]. Dietetic management and intestinal recovery are key to coeliac disease care, but self‐management also carries psychosocial demands [10].

In recognition of the demands of self‐management, international agendas call for more integrated approaches to mental and physical health in long‐term conditions [11]. In coeliac disease, clinical guidance and service models vary by region and focus largely on diagnosis, dietary education, serology, nutritional status, and bone health [1], with limited guidance on addressing psychosocial challenges in routine care. Service pressures are likely to contribute to this gap. Not all people with coeliac disease will see a healthcare professional, and when they do, appointments may be brief, and typically prioritise the GFD and intestinal recovery [12]. While psychosocial challenges may be recognised, patients describe unmet emotional needs and a desire for more emotional support than is routinely available [13]. Several interventions have sought to address specific psychological aspects of living with coeliac disease. These include supportive psychological counselling for adults with coeliac disease and affective disorders [14], group education to improve psychological wellbeing [15], and behavioural interventions designed to improve GFD management in adults [16, 17]. While these studies demonstrate that behavioural and psychological support may improve patient outcomes, they have largely been evaluated as standalone research interventions rather than integrated, routinely available models of adult coeliac disease care. Consequently, adults with coeliac disease continue to have limited access to integrated behavioural and psychological support following diagnosis.

The aim of this project was to co‐develop an evidence‐based support framework to strengthen GFD management and support wellbeing among adults with coeliac disease. We did not intend to develop or evaluate a behavioural intervention, but to specify support functions required to address behavioural and psychosocial needs, providing a foundation for future intervention development and implementation. Co‐design ensured that the framework was grounded in lived experience, clinically feasible, and sensitive to the practical, cultural, and emotional aspects of managing a GFD. The specific objectives were to:

1. Identify key aspects of GFD management and psychosocial support needs to inform a joined‐up dietetic and psychological support framework for adults with coeliac disease;
2. Use theory to clarify how different elements of the framework were expected to work together to support GFD management and wellbeing.

## 2 Materials and Methods

### 2.1 Design

A co‐design approach was used to develop the GLOW framework [18]. The work was informed by a constructivist perspective, drawing on both lived and professional experience. Discussions focused on shared meaning‐making and practical fit for routine care.

### 2.2 Team and Setting

The team, including a lived experience researcher, was supported by a Project Advisory Panel, which included a psychologist specialising in dietary‐managed conditions, two specialist coeliac disease dietitians, a gastroenterologist, representation from Coeliac UK (the national charity for coeliac disease), and two lived experience advisors. The panel met quarterly throughout the 18‐month project, contributing perspectives that influenced study design, interpretation of findings, and development of the GLOW framework (Table 1).

**Table 1:** Role of the project advisory panel across the research process.

**Stage**	**Panel input**	**Impact on study**
Protocol design	Recommended discussion guides draw on lived experience and remain evidence based. Advised separate early sessions for patients and providers to mitigate power imbalances.	Discussion guides developed and piloted with panel. After discussion, final design included separate Workshops 1‐2 and a joint Workshop 3.
Recruitment	Discussed recruiting through Coeliac UK versus the National Health Service. Researchers worried Coeliac UK recruitment might mean under‐represented groups could be missed. Panel suggested purposive outreach to specific Coeliac UK groups to balance this. Discussed who counts as “under‐represented,” identifying South Asian communities, men, and adults > 65 years. Agreed to prioritise early recruitment of these groups, recognising it would take more resource. Explored including people with learning difficulties but concluded this would require bespoke adaptation; group not excluded but not actively targeted. Noted that initial provider sample did not reflect National Health Service staff diversity. Advised efforts to recruit from primary care and community roles. Lived‐experience members commented that General Practitioners rarely feature post‐diagnosis and suggested approaching practice nurses instead.	Recruitment began with purposive outreach to under‐represented communities. Sampling order prioritised these participants to allow time for engagement. Criteria for under‐representation were documented to guide recruitment. Additional recruitment waves targeted National Health Service providers via regional dietetic networks, primary‐care mailing lists, and community practice staff (social prescribers, nurses). Resulted in a mixed‐expertise provider sample.
Data collection	Highlighted need for visible coeliac voice in facilitation and coding. Reviewed early workshop summaries and suggested additional prompts for underexplored areas (e.g. cultural influences).	Lived‐experience researcher co‐facilitated workshops and co‐analysed data. Panel feedback prompted additional prompts on cultural influences in later sessions.
Analysis	Emphasised the importance of including the coeliac voice in data analysis and training for lived‐experience team members.	Lived‐experience researcher contributed to coding and interpretation. Preliminary themes discussed in Workshop 2 for participant refinement and brought back to Project Advisory Panel.
Framework development	Proposed that the framework name convey optimism and hope and suggested “GLOW” (Gluten‐free Living Optimised Wellbeing). Recommended avoiding “psychology” in the title to prevent narrowing scope. Emphasised that the framework should validate the real challenges of living gluten‐free and situate behaviour change within people's everyday contexts.	Framework named GLOW, reflecting a positive and inclusive framing. Language and visual design focused on compassion, validation, and realism rather than idealised adherence. Framework content explicitly integrates recognition of social and contextual challenges.
Dissemination	Encouraged accessible dissemination to coeliac communities and offered to co‐present findings.	Findings presented at gluten‐free cafés and community support groups; accessible summary materials and visual framework tools developed, co‐authored by lived‐experience panel members, for public dissemination.

### 2.3 Theoretical Framework

Development was guided by the Theory of Planned Behaviour (TPB) [19] and Acceptance and Commitment Therapy (ACT) [20]. TPB and ACT were used to support the design of workshop prompts and to guide interpretation of stakeholder accounts, and were not introduced explicitly to participants.

TPB was selected because of its previous use in coeliac disease [21], providing continuity with existing evidence and supporting translation into an applied support framework relevant to routine care. TPB was used to help structure discussions about GFD management as a behaviour influenced by social expectations, perceived capability, and responsibility, and to interpret how patients and healthcare professionals understood confidence, control, and social expectations around managing a GFD. ACT was used to complement TPB and to support interpretation of the emotional demands of GFD management under conditions of environmental uncertainty. The use of ACT was informed by co‐production, with lived experience contributors prioritising emotional adaptation, avoidance of valued activities, and the challenge of living well despite persistent risk. ACT concepts, including psychological flexibility and values‐based action, aligned closely with these priorities and helped make sense of patterns described by participants. Together, TPB and ACT provided a sensitising lens through which both behavioural and psychosocial aspects of lifelong GFD management could be explored.

### 2.4 Participants and Recruitment

Eligible participants were: (a) adults (≥ 18 years) with a diagnosis of coeliac disease, and (b) healthcare professionals working with adults with coeliac disease in the United Kingdom across primary and secondary care. Patients currently under investigation for, or diagnosed with, refractory coeliac disease were excluded due to substantially different care needs [22], as were providers working solely in paediatric settings. Internet access and electronic informed consent were required.

Recruitment began through the National Health Service. Healthcare providers were also recruited via professional networks (e.g. British Dietetic Association, British Psychological Society, British Society of Gastroenterology). Interested individuals accessed study information through an email with an embedded recruitment poster. They were directed to an online information sheet and invited to contact the research team, who answered any questions by email or telephone, prior to providing electronic consent.

To reach underserved patient groups, recruitment was extended through Coeliac UK networks and community organisations, including cultural and faith‐based venues, community centres and voluntary sector health and wellbeing groups. Underserved patient groups were identified by our Project Advisory Panel as South Asian communities, adults aged > 65 years, and men. A maximum‐variation sampling strategy enabled us to capture a wide range of demographic, professional, and geographical perspectives.

Sample size was guided by the principles of information power [23]. Recruitment of ~30 participants was planned but expanded to at least 50 to support diversity of perspectives and depth of data. This allowed inclusion of a broad range of patient and provider experiences.

### 2.5 Procedure

Following consent, participants were invited to join three online co‐design workshops. Those unable to attend one or more workshops, or preferring an alternative format, were offered a one‐to‐one interview. Individual interviews covered equivalent content to Workshops 1 and 2, enabling participants unable to attend group sessions to contribute to the same stages of framework development. Each workshop lasted approximately 150 minutes (including a 15‐minute break), involved 3‐8 participants, and was facilitated by researchers without prior relationships with participants. Workshops 1 and 2 were held separately for patients and providers; workshop 3 brought both groups together.

Sessions were conducted via Microsoft Teams and audio‐recorded for transcription. Discussions were visually mapped in real time using online collaboration boards, hosted by Miro. Table 2 summarises the purpose and activities of each workshop, which included:

1. Mapping experiences and identifying challenges (separate patient and HCP groups) to build a multi‐perspective understanding of gluten‐free living, psychological wellbeing, and current care.
2. Reducing burden and generating solutions (separate groups) to move from challenges to potential solutions. Prompts focused on framework‐relevant questions (e.g. What services could reduce this burden? What has helped before? What can providers realistically deliver? What support would make the most difference?).
3. Joint review and refinement of GLOW (mixed groups) in which patients and providers reviewed a draft framework outlining components, behavioural and psychological targets, resource requirements, and intended outcomes, and provided structured critique to refine accuracy, feasibility, and acceptability.

**Table 2:** Overview of co‐design workshops with patients and providers.

Workshop	Purpose	Activities
1. Mapping experiences and identifying challenges *(patients and providers separately)*	To build a contextualised, multi‐perspective understanding of experiences related to gluten‐free living, psychological wellbeing, and current care frameworks.	Experience‐mapping activity across four quadrants: Think/Feel, See, Hear, Say/Do.
		Group identification of Pains (barriers, fears, difficulties) and Gains (needs, hopes, preferred forms of support).
		Facilitated discussion and clustering of themes on Miro.
2. Reducing burden and generating solutions *(patients and providers separately)*	To transition from understanding challenges to co‐developing potential support strategies and framework components.	Presentation of experience map developed across all workshop 1 participants.
		Introduction to “Capacity Bucket” metaphor. Identification of bucket “fillers” (stressors, burdens) and “taps” (supports that reduce load). Small‐group brainstorming on feasible supports, skills, services. Prioritisation of promising ideas and list of candidate framework components.
		Resource considerations and feasibility constraints.
3. Joint review and refinement of GLOW *(mixed groups)*	To integrate and negotiate perspectives across patient and provider groups, refining the draft framework for accuracy, feasibility, and acceptability.	Walk‐through of first draft of GLOW.
		Structured appraisal across: Accuracy, Feasibility, Acceptability, Clarity, Completeness.
		Final prioritisation and agreement: “Is this deliverable in practice?”, “Does this feel supportive, empowering, and sustainable?”, “Are any steps, resources, or transitions missing?”

For participants choosing individual interviews, the content of Workshops 1 and 2 was condensed into a single 90‐min session. Interview participants were invited to join Workshop 3 or provide email feedback. Adults with coeliac disease received a £25 voucher for each workshop or interview. Withdrawal from the study did not affect reimbursement.

### 2.6 Data Analysis

Data were analysed using an iterative framework approach [24]. Analysis was interpretive and collaborative, with attention to how meanings were shaped through lived and professional perspectives (see positionality statement, Appendix 1). The aim was to bring participant accounts together to inform the development of a workable, theoretically informed support framework grounded in diverse experiences of coeliac disease care.

Workshop and interview recordings were transcribed professionally and reviewed alongside collaboration boards and field notes collected by four researchers. Using inductive open coding, the team captured emotional, behavioural, relational, and contextual aspects of living with or supporting coeliac disease. Coding was discussed reflexively, with multiple interpretations retained. Inductive codes were then examined using sensitising concepts drawn from the TPB and ACT to deepen interpretation. This supported exploration of how experiences related to confidence, responsibility, social expectations, emotional adaptation, and coping with ongoing risk. Through team discussion, codes were organised into an analytic framework spanning behavioural aspects of GFD management, psychosocial demands, contextual influences, and service‐related needs. Framework matrices allowed comparison across participant groups, workshop stages, and settings, supporting identification of patterns as well as points of difference. These matrices informed the development of interpretive themes, which were translated into candidate GLOW framework components by examining the challenges described, the forms of support implied, and the resources or actions required within routine care.

Themes and framework components were drawn as a preliminary logic model outlining anticipated links between support elements and outcomes. This model informed the draft framework reviewed in Workshop 3. The refined framework was then shared for wider feedback through Coeliac UK support groups and community events, with structured feedback reviewed collaboratively with lived experience contributors and the Project Advisory Panel to ensure credibility, relevance, and contextual fit. Table 3 provides a step‐by‐step summary of the analysis.

**Table 3:** Overview of the reflexive, iterative framework analysis.

Analytical stage	Application
Familiarisation	Four researchers (RS, SH, AS, AP) read transcripts and reviewed Miro boards/notes. Initial reflections on patterns, surprises, and positionality were shared in team discussions.
Inductive open coding	Generated inductive codes capturing emotional, behavioural, relational, and contextual aspects of gluten‐free living and care. Interpretations were discussed reflexively, valuing multiple possible readings.
Theory informed sense‐making	TPB and ACT were used as sensitising lenses to deepen interpretation of inductive codes, highlighting behavioural and psychological processes without constraining meaning.
Developing the analytical framework	Codes were organised into a working framework across behavioural targets, psychological processes, contextual influences, and service‐related needs. Matrices enabled comparison across groups, workshop stages, and theoretical constructs.
Theme development	Framework matrices were interpreted to develop themes capturing behavioural, psychological, and system‐level needs shaping GFD management and wellbeing. Divergent perspectives were kept visible rather than collapsed.
Translating themes into GLOW framework components	Themes were mapped to candidate framework components by identifying: the problems they addressed, behaviours targeted, psychological processes supported and required resources or professional actions.
Building the preliminary logic model	Themes and components were integrated into a logic model detailing hypothesised processes of change and anticipated outcomes. This informed the draft framework presented in Workshop 3.
Public refinement	The refined framework was shared at gluten‐free cafés and Coeliac UK groups. Structured feedback was reviewed collaboratively with the Project Advisory Panel to ensure credibility, relevance, and contextual grounding.

Reporting was guided by the Guidance for Reporting Involvement of Patients and the Public 2 reporting checklist (Appendix 1) [25]. Participant quotes are attributed by participant group rather than role or demographic characteristics to minimise the risk of deductive disclosure, given small numbers in some groups. Analytically relevant contextual differences are described in the text.

## 3 Ethical Approval

Ethical approval was granted by the Health Research Authority and the East of Scotland Research Ethics Service (24/ES/0016).

## 4 Results

### 4.1 Participants

Of 63 participants who provided consent, 62 took part in the co‐design process; 35 adults with coeliac disease and 27 healthcare providers. Most providers were dietitians (n = 20, 74.1%), alongside a gastroenterologist, two general practitioners, two practice nurses, a psychologist, and a pharmacist. Patients had a mean age of 49 years (SD = 19·1, range 18‐78). Participants with coeliac disease reflected a broad range of backgrounds: nine from global majority ethnic backgrounds, eleven from lower income households, and ten aged over 65 years. A detailed summary is provided in Table 4.

**Table 4:** Participant characteristics and data collection overview.

	Adults with coeliac disease (N = 35)	Healthcare providers (N = 27)
Demographics		
Mean age (SD), years	49.0 (19.1)	40.8 (12.8)
Age range, years	18‐78	27‐62
Gender (% female)	24 (68.6)	21 (77.8)
Ethnicity (%)		
White	26 (74.3)	21 (77.8)
Asian or Asian British	6 (17.1)	3 (11.1)
Black or Black British	2 (5.7)	1 (3.7)
Other ethnic group	1 (2.9)	2 (7.4)
Household income (%)^a^		
Lower income	11 (31.4)	—
Middle income	8 (22.9)	—
Higher income	16 (45.7)	—
Healthcare provider role (%)		
Dietitian	—	20 (74.1)
Gastroenterologist	—	1 (3.7)
General practitioner	—	2 (7.4)
Practice nurse	—	2 (7.4)
Psychologist	—	1 (3.7)
Pharmacist	—	1 (3.7)
Data collection participation (%)^b^		
Completed one‐to‐one interview	19 (54.3)	14 (51.9)
Attended Workshop 1	16 (45.7)	13 (48.1)
Attended Workshop 2	11 (31.4)	11 (40.7)
Attended Workshop 3	27 (77.1)	18 (66.7)

^a^ Total household income was self‐reported and categorised into lower (<£30,000), middle (£30,000–£59,999), and higher (≥£60,000) income groups.

^b^ Participants could take part in Workshops 1–3 and/or complete a one‐to‐one interview. Some participants who completed an interview also participated in Workshop 3; participation categories are therefore not mutually exclusive.

Engagement varied across study stages, 29 participants (46.8%) attended Workshop 1, 22 (35.5%) attended Workshop 2, 33 (53.2%) completed a one‐to‐one interview, and 45 (72.6%) participated in Workshop 3.

Analysis developed three themes, which informed the co‐developed GLOW framework (Table 5).

**Table 5:** Overview of themes and implications for the GLOW framework.

Theme	Subtheme	Definition	Illustrative quotes	Implications for GLOW framework
Theme 1. A fragmented healthcare landscape that unsettles expectations, confidence and support	1.1 Diagnosis as either orientation or unguided adjustment	Diagnostic encounters served as early meaning‐making points. Some grounded people with clear explanations, referrals and emotional space; others left individuals confused, unsupported, or unsure how to proceed. These early experiences shaped expectations, confidence, and legitimacy in seeking help.	*They literally phoned me when I was in class and said‐ They just said, “Oh, yeah. We think you've got Coeliac Disease.” Not even like really gave me an explanation. I had to say, “Well, what is that then?” and I had to then Google in the back of my classroom like, what is this?* [Patient] *I had at least two workshops with a consultant dietitian who was particularly interested and experienced in coeliac… and a follow up one. I've had at least an annual checkup with her, and I've also used the Coeliac Society dietitians for queries and I feel I've been really well supported*. [Patient] *You would explain about the long‐term consequences and risks of not adhering to the diet… try and gain an idea of their understanding of what foods contain gluten… And then I would provide them with like information leaflets and then refer them on to a dietitian if they were struggling for more in‐depth type of support*. [Provider] *I think they only focus on the physical side of things. They never asked me about, “Oh, how are you feeling after the diagnosis,” or, “These are the sorts of challenges you face, and this is how you can manage later on”* [Patient].	Early diagnostic encounters created either clarity or confusion, shaping expectations, emotional adjustment and perceived capability. GLOW includes a consistent, psychologically informed orientation stage to replace guesswork with clear foundations.
	1.2 Discovering variation: comparison that generates self‐doubt and collective motivation	Workshops revealed wide variation in follow‐up, prescriptions, and dietary support. Comparing experiences produced surprise, frustration, and questions about whether people had “missed something.” Providers also recognised inconsistencies and expressed motivation for shared standards. Social comparison shaped confidence, capability and expectations of care.	*Participant 1: I haven't had an appointment to‐ to see a dietitian or got a yearly blood test thing*. *Participant 2: Sorry?* *Participant 1: I haven't got a yearly blood test thing. I've never had that*. *Participant 3: No, me neither. I will ask for that*. *Participant 1: It makes me wonder whether I've forgotten something, or, or if I'm doing something wrong*. [Patient Workshop] *To be honest, I've never actually asked if I can have face to face and maybe that is something I should do. I just didn't want to be‐ I'm too pressuring for them*. [Patient] *It's interesting… I didn't realise quite how different it was even within places that did have services or specific protocols*. [Provider] *It's nice to have the time to reflect on what you're doing and see there are things you're doing well, and then the things you can then improve on from what other teams are doing*. [Provider]	Variation in services influenced confidence and role expectations for patients and providers. GLOW establishes shared support functions and aligned messages to reduce inconsistency across regions and settings.
	1.3 Compensatory strategies and their unintended consequences	In the absence of consistent support, patients and providers turned to social media, peer groups, self‐learning and experimentation. These strategies offered reassurance but also introduced misinformation, emotional labour and additional cognitive load as individuals tried to create coherent guidance themselves	*I've joined various groups online and stuff but if I'm honest, they haven't been very helpful because‐ I don't know. Some of the information is misleading, or they don't know what they're really talking about and they share like false information*. [Patient]*Rather than just saying like, “You need to self‐manage your condition now.” I think we're trying to think of, what's that bridge of kind of what's the gap. So I'm developing a resource that kind of has all of those kind of links that we've been discussing, so patients can kind of access information and hopefully in a trusted way rather than kind of going on to Google and getting lost there*. [Provider] *They've just moved, they're out of area, or patients who‐ you know, were diagnosed like a few years ago, have never seen a dietitian. They come in and that is a lot harder consultation because rather than educating the patient, you're almost trying to deconstruct all of their negative beliefs or wrong beliefs*. [Provider] *It seems to be a lot of my self‐directed learning and you're just hoping that you're doing the right thing. It feels a bit experimental to be like I read this, I might find this useful, I'll try it out*. [Provider]	Reliance on unregulated information impacts cognitive load and misinformation. GLOW provides centralised, coeliac‐specific, evidence‐informed resources to stabilise messaging and reduce avoidable emotional labour.
	1.4 Clinical dilemmas without shared guidance	Providers struggled with how to communicate dietary strictness, how far to explore emotional or psychological concerns, and when to refer. Without shared frameworks, this led to inconsistent messaging, role uncertainty, and variability in how distress or risk was handled.	*So some patients I will say right what could be our small goal for the next four to six weeks, is it that you know, not everything is going to be gluten free. But ultimately we would want everything gluten free*. [Provider] *We can obviously advise on the behaviour but we then sort of struggle to perhaps have the knowledge and the confidence to delve more in to the health psychology around why they're not sort of following that behaviour*. [Provider] *I had an autistic young person who again wasn't following the diet. We focused on one meal at a time, can we make this meal gluten free because sometimes people find it overwhelming I think if the whole diet has to be gluten free forever. So I have found sometimes that people benefit from breaking it down into, to chunks*. [Provider] *And I guess being devil's advocate if we say that once or twice a year is okay then does that slide to be, oh well you know, it's now three or four times a year or once a week or once a week is okay, then more often*. [Provider] *It's not about trying to get zero gluten in your diet for life because that's never going to happen. It's just about mitigating risks and you being happy with that*. [Provider]	Uncertainty about strictness messaging, emotional support, and referral routes highlighted the need for clearer frameworks. GLOW specifies behavioural principles, psychologically informed communication practices, and clearer frameworks for stepped psychological support.
Theme 2. Reconstructing a gluten‐free identity: how values, legitimacy, and life‐stage transitions shape support needs	2.1 Identity reconstruction as value‐driven	Adjusting to coeliac disease required people to rebuild routines in ways that protected cultural identity, family roles, and personal values over time. Given food's social and cultural significance, adaptation was often about preserving belonging, meaning and continuity, not just avoiding gluten.	*I think it's picking these battles and saying right, what could work in this instance for this patient? Because I always say that, you know, the gluten free diet never gets implemented in the same way*. [Provider] *Being an Asian woman… it's considered extremely rude not to eat what you're offered… that's going to be really problematic for me*. [Patient] *I'd say that my biggest thing I'm proud about is it's‐ It kind of forced me to become like a cook and a baker. And I now love cooking and I love baking. … It feels really nice because it's actually now something that I enjoy. And that's come from having coeliac*. [Patient].	Adaptation reflected cultural values, roles, and belonging. GLOW incorporates culturally relevant examples and supports alignment between gluten‐free living and personally meaningful identities.
	2.2 Misrecognition as an emotional‐identity threat	Patients described frequent experiences of being dismissed, trivialised or misinterpreted as “fussy,” “attention‐seeking,” or part of a lifestyle trend. These moments of misrecognition threatened identity, undermined legitimacy, and shaped emotional and behavioural responses to eating in social spaces. Providers also observed the impact of stigma on dietary management.	*I didn't want to say, ‘Have you remembered…?’ It feels selfish. It's awkward sometimes*. [Patient] *It's like you're making a fuss… ‘It's not going to kill you.’ Well, not today, but long term it will*. [Patient] *I have friends that sometimes as well like, they feel like they really want to include me so they'll plan ahead and make these wonderful plans and everything and then I'm like, I'm not your child. I want to be an adult. I want to be able to make my own decision, you know?* [Patient] *We see partners that don't support their family… you can never implement a diet in a vacuum. Sometimes there's all these barriers from the social context that put more pressure and make it more difficult*. [Provider]	Stigma and misunderstanding impacted patient confidence and self‐advocacy. GLOW embeds strategies that support safe disclosure, reinforce legitimacy, and address the emotional and social impacts of misrecognition.
	2.3 Life‐stage transitions and ongoing adjustment	Transitions such as university, work, travel, dating, pregnancy and ageing required repeated re‐negotiation of gluten‐free living. People described shifting emotional responses, evolving practical strategies, and renewed vulnerabilities across the life course.	*One of the things we're going to try to work on is transition. So we know at this age, adherence can be really difficult, you know. It's all about forming identity. Many of these patients don't want to be different and also, a bit older, going to university*. [Provider] *Young adults dating… they feel they can't be upfront because they'll look fussy*. [Provider] *I could've done with psychological support before uni… living with six people in a shared kitchen was terrifying*. [Patient] *The sort of situation where somebody has a very ill member of their family, or they have a bereavement, or divorce, or something very traumatic and maybe they just don't prioritise their own wellbeing in general, but probably also the gluten free diet. So I think you can have that sort of situation, where a very distressed person, it just isn't their priority then*. [Provider]	Transitions repeatedly destabilised confidence and routines. GLOW includes anticipatory guidance and re‐enterable support to help people navigate challenges across the life course.
Theme 3. Gluten‐free management as lifelong psychological work	3.1 Cognitive labour and fluctuating capacity	Managing coeliac disease required continuous cognitive work: planning, monitoring, label‐reading, anticipating risk, and adjusting to fluctuating emotional or practical capacity. Providers described using pacing and stepwise strategies to avoid overwhelming people.	*They have gluten free menus but it doesn't necessarily mean that their coeliac safe as well. So I think that's obviously something where you've got to ask a lot because of contamination and things like that and also, yeah just checking the back of everything, making sure‐ It's just a lot of control. In terms of psychology you've got to think about every aspect of it before you actually consume something, whereas somebody else could probably just indulge in something* [Patient] *It's actually quite exhausting having to think every time you even pick something up […] ‘is this okay?’* [Patient] *There are a few people that then I say, ‘Well let's do this one thing at a time… just look at breakfast… then cupboards… then the shops… one thing each week*. [Provider]	GLOW incorporates capacity‐matched planning tools, simplification strategies, and flexible support options that adjust to fluctuating cognitive bandwidth.
	3.2 Navigating risk and uncertainty as an inherent psychological task	Beyond routine planning, patients described ongoing negotiation of risk and uncertainty as a distinct psychological task. Eating out, travelling, or relying on others to prepare food triggered hypervigilance, fear of contamination, and confidence “wobbles,” particularly after breaches in supposedly safe settings. Some withdrew from valued activities to avoid risk. Providers recognised these patterns and used reassurance, graded exposure and risk‐assessment conversations to help people maintain engagement while acknowledging that zero risk is unattainable.	*It excludes you sometimes. It makes you feel a bit isolated. It makes you, sometimes you'll be like, “Oh, I just won't go because it's too much of a hassle and I don't want to tell people to change their plans.” It can sort of‐ like I say, you get anxiety around things to a lesser or greater extent*. [Patient] *Be as cautious as you can… but not to say, ‘I'm never going to eat out again.’… if people are very anxious… I encourage them that if they make an occasional mistake… they shouldn't be beating themselves up*. [Provider] I spoke to a lady yesterday who, you know, had really good adherence habits and then I asked her a bit about, “Okay, how do you navigate eating out?” She said, “I don't eat out just because I am just fatigued from all the asking questions.” They associate eating out with an experience that's not enjoyable really. [Provider]	GLOW provides structured strategies for graded exposure, confidence‐building, and shared risk calibration to help people tolerate uncertainty and sustain participation in valued activities.
	3.3 Distress as common, variable and often hidden	Emotional distress was widespread but variably expressed. Patients described frustration, sadness, anxiety about contamination, restrictive eating patterns, and marked lows following gluten exposure. Many were unsure whether their struggles “counted,” contributing to under‐disclosure in appointments. Providers recognised distress but felt constrained by time, confidence and unclear referral routes, sometimes worrying about uncovering needs they could not contain. Distress was described as cyclical, intensifying during stressful periods or after accidental exposure.	*If you already have the sort of like control relationship with food, it sort of adds to that. So in a way, that sort of adds to the psychological side of it and I really haven't been able to get any support with that element of it*. [Patient] *I feel like I probably should be diagnosed with ARFID. But if there's no support, what's the point? I've fought all my life, and it's never been connected to my coeliac disease*. [Patient] *So we've had that feeling there's a big spectrum of the patients, so there's one end that struggle and are very anxious and need support, there's a pot in the middle that are kind of well and manage it and then there's the other end that don't engage and don't comply*. [Provider]. *We often end up focusing on the abdominal pain, the loose stools, the weight loss and less about the chronic fatigue which means that they can't go to work. The non‐specific other symptoms that they don't tell anybody about, psychosocial factors, or things that patients won't report to you, unless you really ask about them*. [Provider] *I'd say a lot of people do find it challenging and yet if you didn't ask, you wouldn't know and you would just focus on are you having gluten? Your bloods are fine. We aren't discussing it so they aren't discussing it*. [Provider]	GLOW embeds routine emotional check‐ins, validation, and mechanisms to address distress early, with clearer referral routes and psychologically informed communication that makes disclosure feel legitimate.
	3.4 The need for structured psychological safety	Both patients and providers highlighted the need for safe, structured spaces to discuss emotional, relational and behavioural aspects of coeliac disease. Patients often avoided burdening peers or providers, while clinicians feared “opening the floodgates” without frameworks for containment. Shared, facilitated spaces were seen as stabilising.	*If I'm meeting people with coeliac, I'm not going to dump everything on them. But a group led by a dietitian or psychologist […] where you can share and learn coping mechanisms, feels safe*. [Patient] *Giving people permission to say, ‘This sucks, I'm annoyed,’ and letting it out in a group or one‐to‐one is really important. It's rubbish […] but then we work out what to do next*. [Provider] *We do need more support and more training when it comes to that emotional side of it. I know we're looking at hopefully developing resources in terms of incorporating that emotional aspect and that behaviour change rather than just here's gluten, eat it or don't eat it… I keep trying to look for even just a webinar or a course or something online and there doesn't seem to be anything*. [Provider] *I think the three key pillars are dietitian, gastroenterologist and psychologist. But all of them need to have adequate expertise in coeliac disease. It's not just the job title. It's the same as, you know. Psychology, general psychology might not understand all of these things with coeliac disease*. [Provider]	Participants valued professionally contained spaces but lacked consistent access. GLOW incorporates psychologically informed interaction principles and clearer referral routes to specialist input where appropriate.

### 4.2 Theme 1. A Fragmented Healthcare Landscape That Unsettles Expectations, Confidence, and Support

Participants described considerable variation in coeliac disease healthcare, from diagnosis to follow‐up, that shaped how they understood coeliac disease and their expectations around GFD management. This fragmentation extended beyond healthcare into online communities, family life, and other settings, meaning individuals often constructed their own frameworks for “safe enough” eating. Theme 1 shows that clarity, consistency, and psychologically informed scaffolding across care frameworks are important to address confusion, emotional burden, and compensatory work created by a fragmented system.

#### 4.2.1 Diagnosis As Either Orientation or Unguided Adjustment

Patients described diagnosis as either an orienting experience, marked by clear explanations and timely referral to knowledgeable providers, or as an abrupt, ambiguous experience that left them to “*work it out*” alone:

They literally phoned me when I was in class and said… ‘Oh, yeah. We think you've got CD.’ […] I had to Google in the back of my classroom like, what is this?

[Patient]

Practical information about the GFD was valued, helping some feel prepared for food choices. However, psychological and emotional aspects of lifelong adjustment were often not directly addressed: “*no‐one has ever asked me how I've felt* [Patient].” Without a guiding narrative, patients relied on guesswork, internet searches, and others’ experiences. 'These early efforts shaped understanding of coeliac disease, and further support felt legitimate. These understandings were revisited over time as patients encountered new information (“*I thought I should get on with it. I'd seen bits on social media about dietitians, but I guess I thought that wasn't really allowed for me*” [Patient]), although early impressions appeared to set the tone for later confidence and expectations of support.

#### 4.2.2 Discovering Variation: Comparison That Generates Self‐Doubt and Collective Motivation

The Workshops made healthcare variation visible, prompting reinterpretation of personal experiences. Many patients were surprised to learn that others had dietetic support or other forms of structured follow‐up, leading some to question whether they had “*missed something* [Patient]”, or whether they were “*doing the GFD right* [Patient].” These comparisons raised questions about what could reasonably be expected from healthcare: “*It makes me wonder whether I've forgotten something, or if I'm doing something wrong* [Patient].” Providers also encountered unfamiliar models of healthcare. Hearing colleagues describe different approaches was both reassuring and unsettling, generating motivation for more aligned standards and ongoing peer dialogue:

It was completely different services. It was nice to be reassured things that are going well but also thinking about other things that we could do within our service. It would be nice to almost have a bit of a network to join in and share.

[Provider]

Comparison appeared to shape how strict patients believed they should be, how capable they felt, and how providers understood their own roles within coeliac disease care.

#### 4.2.3 Compensatory Strategies and Their Unintended Consequences

In the absence of consistent structures, participants described compensating for gaps in healthcare. Patients turned to social media, blogs, charities, and informal groups to make sense of gluten‐free living. While these sources offered validation and practical strategies, they also introduced conflicting messages that were not always aligned with clinically verified guidance, requiring providers to correct this misinformation: “*Most of my education around Coeliac Disease is coming from like Instagram, right? … There's a weird amount of things that is not given to you as information by doctor* [Patient]”. Providers, meanwhile, described similar compensatory actions, seeking out their own training, relying on ad‐hoc peer learning, or using intuition to address psychological and behavioural needs they felt ill‐equipped to manage:

It feels a bit experimental to be like, I read this, I might find this useful, I'll try it out.

[Provider]

These efforts were meaningful attempts to create coherence in an incoherent system, yet they introduced conflicting messages, heightened responsibility, added emotional labour, and safety concerns. Exposure to conflicting advice also created uncertainty about which sources could be trusted, often exacerbating anxiety, and the cognitive and emotional burden of decision‐making. Both patients and providers were left constructing, and sometimes defending, their own interpretations of what good support and “*safe enough* [Provider]” GFD management should look like. They drew variously on social media, blogs, peer advice, or ad hoc training, highlighting a need for coordinated guidance rather than further individual responsibility.

#### 4.2.4 Clinical Dilemmas Without Shared Guidance

Providers described struggling to communicate adherence expectations in ways that balanced safety with wellbeing. Emphasising absolute GFD strictness felt necessary for intestinal healing but risked heightening worry or disengaging patients who were ambivalent or distressed. Conversely, softening the message felt clinically unsafe:

Should we always come across as ‘you have to stick with this 100%’… or say ‘a mistake once or twice won't do long‐term damage’?

[Provider]

This dilemma extended into the emotional aspects of gluten‐free living. While practical questions about food choices and symptoms were routine, providers varied widely in whether, and how, they explored distress, avoidance, shame, or low mood. Some attempted emotional check‐ins using strategies they had picked up informally, yet even those who did so felt uncertain about their remit and worried about uncovering needs they had no clear referral route for:

Have I got somewhere to put them to if this all goes really badly wrong? You don't want to sometimes find out too, too much because then you're left going, I don't know where to put you.

[Provider]

For patients, this inconsistency contributed to uncertainty about whether emotional challenges were appropriate to raise, or whether their difficulties “*counted*.” The lack of shared guidance left participants navigating grey areas around what support was legitimate and how to balance GFD management with psychological wellbeing. The absence of shared guidance meant distress and uncertainty were often recognised but not consistently addressed, reinforcing the need for psychologically informed foundations that validate emotional responses while providing safe behavioural parameters.

### 4.3 Theme 2. Reconstructing a Gluten‐Free Identity: How Values, Legitimacy and Life‐Stage Transitions Shape Support Needs

After diagnosis, patients described the ongoing work of reconstructing a gluten‐free identity that aligned with their cultural values, family roles, and sense of self. Although providers tried to adapt guidance to patients' contexts, the psychological labour of identity reconstruction largely fell to patients. Theme 2 shows that gluten‐free living requires sustained identity reconstruction, to support GFD confidence and management.

#### 4.3.1 Identity Reconstruction As Value‐Driven

Adjustment to coeliac disease went beyond GFD management, extending to preserving continuity with cultural, familial, religious, and personal identities. Food was important to social and cultural practices, woven into routines participants felt they “*could never walk away from* [Patient].” Patients spoke of creatively adapting recipes, maintaining family traditions, or reworking long‐held rituals to avoid losing parts of themselves. Providers recognised these pressures, noting that standard dietary advice often failed to account for culturally specific practices, household expectations, or religious rituals.

Some cultures where it's more family‐orientated… it can be very difficult to refuse food…. It would be seen as extremely rude to say, ‘I can't eat that.’

[Provider]

While many patients found ways to stay connected to valued communities, others described loss, disorientation, and longing when GFD management disrupted important aspects of who they were. For some, the pressure to maintain cultural or social belonging meant occasionally eating gluten despite knowing the risks, reflecting the tension between protecting health and preserving identity.

I'm Italian… not being able to share what's on my plate affects the way I am interacting with people.

[Patient]

#### 4.3.2 Misrecognition as an Emotional‐Identity Threat

Despite efforts to protect valued identities, experiences of misrecognition destabilised this personal work. Patients described a persistent social narrative in which coeliac disease was framed as fussiness, inconvenience, or a lifestyle preference. These interpretations appeared to contribute to shame, reluctance to assert needs, and ongoing thoughts about how others might perceive them. Many avoided asking whether food was safe, withdrew from social eating, or, at times, ate gluten to preserve relationships or avoid appearing demanding.

You don't want to be a gluten‐free princess… This isn't lifestyle, this is key.

[Patient]

Some patients outright refuse to advocate for themselves because they don't want the attention.

[Provider]

Misrecognition posed an emotional and identity threat that shaped behaviour in ways not captured by current framings of GFD management. Providers recognised how these dynamics undermined confidence and self‐advocacy yet noted that current resources offered little support for the moral and relational labour patients were performing.

#### 4.3.3 Life‐Stage Transitions and Ongoing Adjustment

Identity reconstruction was ongoing. Many patients described mixed emotional responses at diagnosis, including relief at having an explanation for symptoms, alongside shock, grief, or disbelief, and a sense that life as they knew it had changed. Even patients who adjusted well recalled early emotions of anger, fear, sadness, or a feeling that the GFD had “*taken something away* [Patient].” These initial reactions represented the first coeliac disruption to identity, as people struggled to reconcile their former selves with the demands of a lifelong dietary change.

I actually felt immediate elation at my diagnosis because I had an answer… But it was when that‐ the dust had kind of settled and then I started to struggle and then I was like, oh my first big trip abroad and, and all the sort of anxiety and stress came out.

[Patient]

As life progressed, further transitions repeatedly unsettled identity. Moving into shared housing, new jobs, dating, travelling abroad, or anticipating ageing and care needs each appeared to reactivate vulnerability. Even those who had coped well for years described re‐emergence of frustration, avoidance, or overwhelm when contexts shifted. Providers recognised these recurring patterns, identifying young adults leaving home as particularly vulnerable, but noted that such transitions were rarely anticipated in current services. Providers were often unsure how to prepare patients for new environments, or how to support safe disclosure:

When they go off to uni or move away, that's where we see the wobble… but we don't really have anything structured to prepare them for that.

[Provider]

These transitions were moments when GFD management had to be reconstructed again, leaving patients temporarily less confident, more avoidant, or more likely to compromise safety to maintain belonging.

### 4.4 Theme 3. Gluten‐Free Management As Lifelong Psychological Work

Reconstructing a gluten‐free identity did not eliminate the ongoing demands of the GFD. Across the lifespan, participants described gluten‐free living as sustained cognitive, emotional, and relational work that remained invisible to others. Self‐management was closely tied to fluctuating capacity; maintaining safety required continuous monitoring, decision‐making, and regulation of distress. Theme 3 shows that gluten‐free living is sustained psychological work, highlighting the need for services to address cognitive load, emotional fluctuation, uncertainty, and containment needs. This requires routine, psychologically informed support with clear guidance for when specialist input is warranted.

#### 4.4.1 Cognitive Labour and Fluctuating Capacity

GFD management required continuous cognitive effort: reading food labels, evaluating risk, planning ahead, carrying backup food, and monitoring cross‐contact in everyday settings. Patients described this work as “*exhausting*” and relentless, particularly when life was busy, unpredictable or emotionally demanding. Several described periods where the cumulative mental load exceeded what they could sustain, making self‐management feel harder even when motivation remained intact. Providers recognised this dynamic, with some emphasising that GFD management often depended on cognitive load:

You've got to have a clear mind to implement this daily […] watch out for cross‐contact, read labels, think ahead. If your mind is cloudy or preoccupied, you simply can't prioritise the diet.

[Patient]

I think a lot of people we see… they are in such a low mood, like so exhausted because they are still having gluten. And actually trying to follow the gluten free diet is really difficult because they are almost in that kind of downward spiral.

[Provider]

Providers described confidence in supporting practical tasks, signposting safe restaurants, suggesting portable foods, offering strategies for shopping on a budget, and connecting patients with local support groups. Some also used behavioural techniques such as pacing, chunking, and structured problem‐solving, and some offered motivational interviewing where training allowed. These strategies aligned support with patients’ fluctuating capacity, yet participants emphasised that practical guidance alone did not resolve the underlying cognitive burden.

#### 4.4.2 Navigating Risk and Uncertainty As an Inherent Psychological Task

Beyond the cognitive load of day‐to‐day planning, patients described navigating uncertainty as a distinct and continuous psychological task. This was compounded by inconsistent and sometimes conflicting information about risk and safety, which undermined confidence in decision‐making. Eating out, travelling, trusting others to cook, or managing unfamiliar environments required ongoing appraisal of risk and vigilance about cross‐contact. Many described a heightened sense of threat, noting that breaches, especially in settings assumed to be “*safe*”, could knock confidence.

If I eat somewhere new, a new restaurant, I would say that in perhaps the next three or 4 h having eaten, I will constantly be kind of self‐monitoring myself. And I will be thinking am I okay? Is my stomach twinging? Am I feeling nauseous? I feel very hyperaware.

[Patient]

Providers recognised this escalation into fear‐driven patterns and described drawing on graded exposure, reassurance, and self‐compassion to help patients maintain involvement in valued activities.

I think we can help them practically and kind of guide them on questions to answer so that they can… assess the risks themselves but ultimately at the end of the day it's still a risk isn't it… the last thing I want to do is twist someone's arm to go out and eat a meal out if that's just really causing them a lot of anxiety.

[Provider]

These accounts highlighted that GFD management required not only knowledge but also the ability to tolerate uncertainty and flexibly evaluate risk.

#### 4.4.3 Distress As Common, Variable and Often Hidden

Emotional distress varied widely. Patients reported frustration, sadness, panic about contamination, restrictive eating patterns, and, for some, marked lows following gluten exposure. Many were unsure whether their struggles “*counted*,” contributing to under‐disclosure in appointments.

I've never really put it to a dietitian because, well I'm the one that's restricting my own diet. But at the same time, if I try and eat something I'm sick. And now I've got the psychological side of it where I think, if I try something and I'm sick, it's going to put me off even more and it sort of‐ It's a bit of a cycle now. So I just have a really limited diet.

[Patient]

Providers recognised these patterns but felt constrained in their ability to respond. Several described mismatches between what patients disclosed in clinic and how psychological services assessed their needs. Individuals who appeared highly distressed were sometimes deemed “*not meeting [*mental health service*] thresholds*.” Patients echoed this, describing referrals that led to assessments where their coeliac disease was “*not understood*.” Without clear referral routes or coeliac disease‐informed psychological input, clinicians worried about missing significant need or opening conversations they could not safely contain.

You don't want to sometimes find out too, too much because then you're left going I don't know where to put you.

[Provider]

Distress was described as cyclical, intensifying during stressful times or after accidental gluten exposure and re‐emerging even years after diagnosis: “*P*s*ychologically, when I get glutened I'm in a really bad place. I've had huge lows with it […] there's nothing consistent about it. It comes and goes depending on what else is happening in my life* [Patient].” This pattern highlighted the need for ongoing forms of emotional support within coeliac disease care.

#### 4.4.4 The Need for Structured Psychological Safety

Participants emphasised the need for structured forms of psychological safety within coeliac disease care. Patients valued professionally facilitated spaces where their reactions would be understood rather than minimised, noting that at times informal peer support did not provide sufficient containment for discussing fear, shame, or distress.

Providers similarly described the importance of psychologically informed frameworks and clear referral frameworks. Several felt uneasy exploring distress without the means to contain it, noting that coeliac disease concerns often fell “*between services*” and were not well understood by general mental health frameworks. This created caution in raising or responding to emotional issues, even when they recognised unmet need.

I wish there were better resources… because there isn't something you can send out and say, ‘We'll discuss it afterwards.’

[Provider]

Importantly, participants also emphasised that psychological referral was not always needed or desired. Many felt that brief, validating interactions, like normalising emotional responses, exploring worry, offering coping strategies, would meet most needs. Providers agreed that, with appropriate tools and training, much early psychological support (e.g. validating and normalising emotional responses and signposting to appropriate resources) could be embedded within routine contacts across settings, including secondary care, primary care, community organisations, or trained voluntary‐sector roles, with specialist mental health provision reserved for more persistent distress.

#### 4.4.5 Co‐Designing the GLOW Framework

The themes informed the development of GLOW, which conceptualises gluten‐free living as ongoing psychological and behavioural work. To accommodate variation in support provision, GLOW specifies core support functions rather than prescribing specific professional roles. Many of these functions can be delivered through psychologically informed interactions across primary care, specialist gastroenterology services, digital interventions, community and charity organisations, and structured peer programmes, with responsibility distributed according to local workforce and infrastructure.

Participants described fluctuating support needs beyond initial diagnosis and limited routes back to care, informing a non‐linear, re‐enterable structure. One patient noted, “*I've got my diagnosis and I'm off in the world but how do I… get support if I need to*?” Participants also differentiated general education, which provides a useful “*baseline*,” from needs requiring personalised input: “*Underlying things… need more of an individual personal way of doing it, and probably more time*.” GLOW therefore separates standardised orientation resources from tailored, capacity‐matched support.

Perceived inequity across regions (described as a “*lottery*”) prompted the definition of minimum support functions applicable across configurations. Psychological needs were considered legitimate but not universal; recognising impact early was valued, although “*an appointment with psychology… for everybody… isn't appropriate*.” This informed a three‐domain structure: (A) Orientation and Adjustment, (B) Sustained Support, and (C) Specialist Psychological Input.

Finally, participants highlighted variation by cultural context and life stage (e.g. “*diagnosed in your 60 s is different than in your 20 s*”). Consequently, contextual tailoring and transition‐specific support were embedded across all domains. The co‐design process identified four core design needs that shaped the final framework:

1. consistent, psychologically informed foundations across settings;
2. strengthened early orientation following diagnosis;
3. visible, aligned expectations about available support; and
4. coordinated, role‐defined care that reduces reliance on individual compensatory strategies.

These needs informed framework structure but do not prescribe where functions are delivered; they can be met within healthcare, digital programmes, community organisations, or structured peer support. Table 6 summarises the domains, mechanisms, and intended outcomes.

**Table 6:** Domains of the GLOW framework: mechanisms, functions and intended outcomes.

Domain	Purpose	Psychological mechanism addressed	Support functions^a^	Anticipated contributions
A. Orientation and Adjustment	Clear orientation that builds early capability, reduces reliance on unregulated sources, and acknowledges identity disruption as part of early adjustment	Early overwhelm; variable access to post‐diagnosis support; identity disruption; misrecognition and legitimacy concerns.	Values‐based framing; orientation that acknowledges emotional adjustment needs; acknowledgement of adjustment; concise correction of misinformation; examples that reflect cultural and social eating practices (e.g. family meals, religious events); involvement of family members or close others in early orientation where appropriate.	Clear, realistic expectations about support, GFD management and living with coeliac disease; reduced reliance on unregulated information for clinical guidance while maintaining peer support; earlier validation of emotional responses; confidence engaging with support frameworks across settings.
B. Sustained Support	Support ongoing adaptation when psychological load exceeds capacity or context changes.	Fluctuating cognitive bandwidth; social identity and belonging; stigma and disclosure barriers; avoidance in response to uncertainty and perceived risk; perfectionism.	Capacity‐matched planning strategies; scripts for navigating disclosure and misrecognition; strategies for managing uncertainty in shared/social eating environments; context‐specific adaptations (travel, work, shared kitchens, cultural events); support during key life transitions (e.g. university, travel); guidance for family members or close others to support safe and sustainable gluten‐free living.	Flexible participation in valued activities; manageable cognitive load; reduced avoidance; culturally congruent adaptations; continued engagement across life changes and fluctuating capacity.
C. Specialist Psychological Input^b^	Provide further support when distress or restrictive eating patterns linked to contamination fears go beyond routine care.	Significant distress related to eating, identity, or social contexts; persistent difficulty engaging with GFD management; restrictive or disordered eating patterns (including risk of eating disorders); anxiety or fear responses; distress affecting functioning or safety.	Psychological formulations that incorporate coeliac disease‐ specific social, identity, and dietary contexts; support for exploring avoidance or withdrawal patterns; assessment and management of disordered eating patterns in the context of coeliac disease; collaborative referral frameworks across healthcare and third‐sector mental health providers; liaison with psychological services to ensure coeliac disease context is understood.	Improved access to psychological support that reflects coeliac disease‐specific context; reduced distress and withdrawal; improved functioning and safety; support aligned with individual values and capacity.

^a^ Functions describe what support must achieve, not which profession or organisation should deliver them.

^b^ Specialist input refers to work delivered by mental health professionals or in collaboration with them.

## 5 Discussion

Healthcare for coeliac disease has centred on GFD education, serological monitoring, and assessment of mucosal healing, reflecting the need to prevent long‐term complications [1]. Given the individual and service burden associated with these complications, there is a rationale for earlier, preventative investment in psychosocial and behavioural support to sustain GFD adherence [10]. Our findings show that managing a GFD also involves sustained psychological, social, and identity‐related work that is not typically addressed in current care frameworks. The co‐designed GLOW framework responds to these challenges by specifying core psychologically‐informed support functions rather than prescribing professional roles, allowing delivery across healthcare, community, digital, and voluntary‐sector settings. By strengthening early orientation, making routes back to support visible, and replacing informal compensatory strategies with coordinated guidance, GLOW shifts attention from information provision alone to the ongoing work required to live well with coeliac disease. Although developed in the United Kingdom, GLOW aligns with global priorities for integrated mental and physical healthcare in long‐term conditions [11] and is designed to be adaptable across different service configurations.

Previous research has highlighted elevated rates of anxiety and depression among adults with coeliac disease [7] and called for greater psychological input in care frameworks [14]. In addition, the European Society for the Study of Coeliac Disease guidelines recommend psychological support primarily for a subset of individuals experiencing distress or coping difficulties [26]. Our findings build on this work by showing that psychological processes, such as identity negotiation, risk appraisal, and cognitive load, shape GFD management even among individuals who described themselves as coping well. This suggests that behavioural and psychological support may need to be offered proactively and embedded into routine care, rather than considered only when distress reaches clinical thresholds. More recently, the independently developed GROW programme described a behavioural telehealth intervention for adolescents with coeliac disease and their caregivers [27]. In contrast, GLOW defines the support functions required for adults to inform future intervention development.

GLOW is intended as a framework rather than a prescriptive intervention. However, the co‐design findings identify several implementation principles that should guide future intervention development. Rather than a one‐off educational intervention, participants described the need for a non‐linear model combining structured support following diagnosis with visible routes back into care as needs change. Support functions may be delivered across healthcare, community, charity and digital settings, with intensity matched to individual need and specialist psychological input available when required. The next step is to operationalise the support functions identified within GLOW and determine how they can be implemented within routine care through further co‐design and feasibility evaluation, before a definitive clinical and cost‐effectiveness trial can be conducted. Existing behaviour‐change models of GFD management have emphasised intention, planning, autonomous motivation, and self‐regulatory skills in GFD management [28, 29]. While these remain important, participants in this study rarely framed difficulties in terms of low motivation. Instead, challenges arose when emotional load, social pressure, or competing life demands reduced capacity to sustain GFD management. Drawing on TPB and ACT helped explain how people sustained the diet under strain, temporarily withdrew from valued activities, or recalibrated what felt ‘safe enough’ in different contexts. These accounts suggest that successful management depends not only on capability, but on the ability to persist when confidence, energy, or social safety are compromised.

This work also identified a pattern similar to long‐term condition burnout. Participants described periods of exhaustion linked to continual vigilance, planning, label reading, and managing others’ responses, sometimes leading to reduced confidence or temporary disengagement from the GFD. This pattern occurred even among those who were knowledgeable and highly motivated. Similar patterns have been described in other self‐managed conditions, including diabetes, where sustained responsibility can lead to emotional depletion despite strong commitment to self‐care [30, 31]. However, burnout as a distinct construct has received limited attention in coeliac disease. Rather than proposing a new diagnostic category, burnout may offer a useful way of understanding when people may need support to restore psychological resources, rather than further education or motivational input.

These experiences were strongly shaped by context. GFD management must happen within sociocultural and structural contexts, including food environments, cultural eating practices, social expectations, and regional variation in healthcare provision, that are not designed for GFD management [5]. Without recognising these challenges, responsibility for adaptation risks being located solely within the individual. GLOW addresses this by incorporating culturally meaningful examples, practical scripts for navigating misrecognition, and anticipatory support around predictable transitions such as leaving home, changing jobs, or travelling. At the same time, it acknowledges that individual behaviour change cannot compensate for unsafe or inequitable environments, and that responsibility for reducing these barriers extends beyond clinical services to community organisations, digital platforms, and the food industry.

Although GLOW shares similarities with holistic long‐term condition models used in diabetes, cardiac rehabilitation and inflammatory bowel disease [32, 33], its organising principles are distinct. Rather than escalating support based on symptoms or adherence alone, GLOW responds to psychological load, identity threat, uncertainty, and contextual disruption. GLOW targets mechanisms identified in this analysis: cognitive capacity, misrecognition, transition vulnerability, risk calibration, and the need for professionally contained psychological support, none of which are explicitly addressed in current coeliac disease frameworks. It represents a process‐oriented extension to biopsychosocial models of dietary self‐management, with flexibility for delivery across clinical and non‐clinical systems, depending on local infrastructure and workforce.

### 5.1 Strengths and Limitations

A strength of this study was sustained involvement of people with lived experience and healthcare professionals throughout the co‐design process. Although we prioritised recruitment of underserved groups and successfully increased representation compared with much coeliac disease research, the sample remained predominantly female, White and relatively affluent. These limitations likely reflect broader challenges in engaging underserved communities and should be addressed in future implementation studies. Many participants were recruited through engaged services or voluntary‐sector networks, and experiences of fragmentation may be greater in less resourced settings. Dietitians comprised most healthcare participants, reflecting current UK models of adult coeliac disease care. Although the study was conducted by a multidisciplinary research team which included expertise in health psychology and long‐term conditions, additional perspectives from practising gastroenterologists and clinicians providing psychological care may have informed future implementation considerations.

Participants willing to attend extended workshops may also have found coeliac disease particularly salient, potentially amplifying the psychological work described. While theoretical lenses supported interpretation, they may have oriented analysis toward psychological and behavioural processes at the expense of structural or economic factors. Finally, GLOW is a proposed framework; its feasibility, cost, and impact require evaluation through pilot implementation and realist testing across diverse settings.

### 5.2 Implications

GLOW's primary contribution is conceptual: reframing GFD management as ongoing psychological work rather than an educational task. While not a clinical manual, the framework provides practical direction by legitimising cognitive load, risk navigation, and identity disruption as routine aspects of care. Many needs identified in this study could be addressed through brief, psychologically informed interactions within primary and secondary care, and community settings, with specialist psychological input reserved for persistent distress. GLOW is intentionally service‐agnostic, allowing functions to be delivered across healthcare, digital platforms, and voluntary‐sector organisations depending on local capacity.

Operationalising GLOW will require investment in psychologically informed skills within existing services, clearer referral frameworks, culturally relevant resources, and tools to support predictable life transitions. These represent adaptations of current provision rather than the creation of new professional roles. Detailed specification of workforce requirements and implementation strategies will be addressed in future work.

## 6 Conclusion

GFD management involves continuous cognitive, emotional and social labour that fluctuates across life stages and environments. Current care frameworks do not adequately address this ongoing work, leaving patients and clinicians to manage gaps informally and inconsistently. The co‐designed GLOW framework offers a grounded, flexible framework for integrating psychological and behavioural support into coeliac disease care. By reframing management as a dynamic, identity‐shaped process rather than a static educational outcome, GLOW offers a foundation for more equitable and person‐centred support.

## Author Contributions

**Rose‐Marie Satherley:** conceptualisation, investigation, funding acquisition, writing – original draft, methodology, validation, visualisation, writing – review and editing, formal analysis, project administration, data curation, supervision, resources. **Shakira Hollyfield:** investigation, methodology, writing – review and editing, project administration, data curation. **Areeba Shahab:** data curation, project administration, methodology, writing – review and editing, investigation. **Angie Pitt:** investigation, writing – review and editing, data curation. **Nick Trott:** funding acquisition, writing – review and editing, supervision. **Lizz Toft:** supervision, writing – review and editing, funding acquisition. **Aftab Ala:** funding acquisition, writing – review and editing, supervision. **Christina J. Jones:** funding acquisition, writing – review and editing, supervision, methodology, investigation, conceptualisation.

## Conflicts of Interest

The authors declare no conflicts of interest.

## Supporting information


Supporting File

## Data Availability

The data that support the findings of this study are available on request from the corresponding author. The data are not publicly available due to privacy or ethical restrictions.

## References

[1] B. Lebwohl , D. S. Sanders , and P. H. R. Green , “Coeliac Disease,” Lancet 391, no. 10115 (2018): 70–81, 10.1016/S0140-6736(17)31796-8. 28760445

[2] J. A. Silvester , I. Comino , L. N. Rigaux , et al., “Exposure Sources, Amounts and Time Course of Gluten Ingestion and Excretion in Patients With Coeliac Disease on a Gluten‐Free Diet,” Alimentary Pharmacology & Therapeutics 52, no. 9 (2020): 1469–1479, 10.1111/apt.16075. PMC7780203 32981131

[3] A. Grabowicz and G. Czaja‐Bulsa , “Misleading Labelling of Gluten‐Free Products in the Light of Eu Regulations: Time for a Change?,” Journal of Consumer Protection and Food Safety 14, no. 1 (2019): 93–95, 10.1007/s00003-019-01208-6.

[4] N. Towers and J. Pratten , “Managing Special Dietary Needs for Food Allergies in the Hospitality Industry; A Survey Based on the Coeliac Disorder,” International Journal of Hospitality Management 22, no. 4 (2003): 341–351, 10.1016/S0278-4319(03)00066-5.

[5] J. A. King , G. G. Kaplan , and J. Godley , “Experiences of Coeliac Disease in a Changing Gluten‐Free Landscape,” Journal of Human Nutrition and Dietetics 32, no. 1 (2019): 72–79, 10.1111/jhn.12597. 30280436

[6] A. Pitt , F. Lerigo , and R. M. Satherley , “Health‐Related Stigma and Challenges Faced by Men Living With Celiac Disease: A Qualitative Analysis,” Journal of the Academy of Nutrition and Dietetics 124 (2023): 674–685, 10.1016/j.jand.2023.11.018. 38040116

[7] E. Clappison , M. Hadjivassiliou , and P. Zis , “Psychiatric Manifestations of Coeliac Disease, a Systematic Review and Meta‐Analysis,” Nutrients 12, no. 1 (2020): 142, 10.3390/nu12010142. PMC7019223 31947912

[8] K. Sainsbury and M. M. Marques , “The Relationship Between Gluten Free Diet Adherence and Depressive Symptoms in Adults With Coeliac Disease: A Systematic Review With Meta‐Analysis,” Appetite 120 (2018): 578–588, 10.1016/j.appet.2017.10.017. 29050807

[9] R. L. Wolf , B. Lebwohl , A. R. Lee , et al., “Hypervigilance to a Gluten‐Free Diet and Decreased Quality of Life in Teenagers and Adults With Celiac Disease,” Digestive Diseases and Sciences 63, no. 6 (2018): 1438–1448, 10.1007/s10620-018-4936-4. 29387990

[10] R. M. Satherley , N. Trott , and A. Ala , “Rethinking Coeliac Disease Care: Why Psychological Understanding Matters,” Lancet Gastroenterol Hepatol 11, no. 6 (2026): 439–441, 10.1016/S2468-1253(26)00020-8. 41831476

[11] Integrating the Response to Mental Health Disorders and Other Chronic Diseases in Health Care Systems, Accessed January 21, 2026, https://www.who.int/publications/i/item/9789241506793;.

[12] M. Nelson , N. Mendoza , and N. McGough , “A Survey of Provision of Dietetic Services for Coeliac Disease in the Uk,” Journal of Human Nutrition and Dietetics 20, no. 5 (2007): 403–411, 10.1111/j.1365-277X.2007.00813.x. 17845374

[13] A. M. Madden , A. M. Riordan , and L. Knowles , “Outcomes in Coeliac Disease: A Qualitative Exploration of Patients’ Views on What They Want to Achieve When Seeing a Dietitian,” Journal of Human Nutrition and Dietetics 29, no. 5 (2016): 607–616, 10.1111/jhn.12378. 27196120

[14] G. Addolorato , G. De Lorenzi , L. Abenavoli , L. Leggio , E. Capristo , and G. Gasbarrini , “Psychological Support Counselling Improves Gluten‐Free Diet Compliance in Coeliac Patients With Affective Disorders,” Alimentary Pharmacology & Therapeutics 20, no. 7 (2004): 777–782, 10.1111/j.1365-2036.2004.02193.x. 15379838

[15] L. Ring Jacobsson , M. Friedrichsen , A. Göransson , and C. Hallert , “Does a Coeliac School Increase Psychological Well‐Being in Women Suffering From Coeliac Disease, Living on a Gluten‐Free Diet?,” Journal of Clinical Nursing 21, no. 5–6 (2012): 766–775, 10.1111/j.1365-2702.2011.03953.x. 22039932

[16] A. J. Dowd , K. T. Y. Tang , M. Y. Chen , et al., “Improvements in Self‐Compassion After An Online Program for Adults With Celiac Disease: Findings From the Power‐C Study,” Self and Identity 22, no. 2 (2023): 197–226, 10.1080/15298868.2022.2074091.

[17] K. Sainsbury , B. Mullan , and L. Sharpe , “A Randomized Controlled Trial of an Online Intervention to Improve Gluten‐Free Diet Adherence in Celiac Disease,” American Journal of Gastroenterology 108, no. 5 (2013): 811–817, 10.1038/ajg.2013.47. 23458849

[18] E. B. N. Sanders and P. J. Stappers , “Co‐Creation and the New Landscapes of Design,” CoDesign 4, no. 1 (2008): 5–18, 10.1080/15710880701875068.

[19] I. Ajzen , “The Theory of Planned Behavior,” Organizational Behavior and Human Decision Processes 50, no. 2 (1991): 179–211, 10.1016/0749-5978(91)90020-T.

[20] S. C. Hayes , J. B. Luoma , F. W. Bond , A. Masuda , and J. Lillis , “Acceptance and Commitment Therapy: Model, Processes and Outcomes,” Behaviour Research and Therapy 44, no. 1 (2006): 1–25, 10.1016/j.brat.2005.06.006. 16300724

[21] K. Sainsbury , B. Mullan , and L. Sharpe , “Predicting Intention and Behaviour Following Participation in a Theory‐Based Intervention to Improve Gluten Free Diet Adherence in Coeliac Disease,” Psychology & Health 30, no. 9 (2015): 1063–1074, 10.1080/08870446.2015.1022548. 25783434

[22] A. Rubio‐Tapia and J. A. Murray , “Classification and Management of Refractory Coeliac Disease,” Gut 59, no. 4 (2010): 547–557, 10.1136/gut.2009.195131. PMC2861306 20332526

[23] K. Malterud , V. D. Siersma , and A. D. Guassora , “Sample Size in Qualitative Interview Studies: Guided by Information Power,” Qualitative Health Research 26, no. 13 (2016): 1753–1760, 10.1177/1049732315617444. 26613970

[24] S. Klingberg , R. E. Stalmeijer , and L. Varpio , “Using Framework Analysis Methods for Qualitative Research: Amee Guide No. 164,” Medical Teacher 46, no. 5 (2024): 603–610, 10.1080/0142159X.2023.2259073. 37734451

[25] S. Staniszewska , J. Brett , I. Simera , et al., “GRIPP2 Reporting Checklists: Tools to Improve Reporting of Patient and Public Involvement in Research,” BMJ 358 (2017): j3453, 10.1136/bmj.j3453. PMC5539518 28768629

[26] A. Al‐Toma , F. Branchi , F. Zingone , et al., “European Society for the Study of Coeliac Disease (ESsCD) 2025 Updated Guidelines on the Diagnosis and Management of Coeliac Disease in Adults,” Part 2: Management, Follow‐Up, and Complex Disease Courses 13, no. 10 (2026): 1855–1886, 10.1002/ueg2.70195. PMC12704582 40999951

[27] J. Vagadori , P. J. Trojanowski , C. Keenan , et al., “Behavioral Telehealth Intervention Development for Adolescents With Celiac Disease and Their Caregivers: The Gluten‐Free Resilience and Overall Wellness (Grow) Project,” Nutrition and Health 32, no. 3 (2026): 869–885, 10.1177/02601060251330938. PMC12771562 40304638

[28] K. Sainsbury , E. P. Halmos , S. Knowles , B. Mullan , and J. A. Tye‐Din , “Maintenance of a Gluten Free Diet in Coeliac Disease: The Roles of Self‐Regulation, Habit, Psychological Resources, Motivation, Support, and Goal Priority,” Appetite 125 (2018): 356–366, 10.1016/j.appet.2018.02.023. 29481913

[29] A. J. Dowd , M. E. Jung , M. Y. Chen , and M. R. Beauchamp , “Prediction of Adherence to a Gluten‐Free Diet Using Protection Motivation Theory Among Adults With Coeliac Disease,” Journal of Human Nutrition and Dietetics 29, no. 3 (2016): 391–398, 10.1111/jhn.12321. 25919067

[30] K. D. Vohs and T. F. Heatherton , “Self‐Regulatory Failure: A Resource‐Depletion Approach,” Psychological Science 11, no. 3 (2000): 249–254, 10.1111/1467-9280.00250. 11273412

[31] F. F. Sniehotta , J. Presseau , and V. Araújo‐Soares , “Time to Retire the Theory of Planned Behaviour,” Health Psychology Review 8, no. 1 (2014): 1–7, 10.1080/17437199.2013.869710. 25053004

[32] N. A. ElSayed , G. Aleppo , V. R. Aroda , et al., “2. Classification and Diagnosis of Diabetes: Standards of Care in Diabetes—2023,” supplement, Diabetes Care 46, no. Suppl 1 (2023): S19–S40, 10.2337/dc23-S002. PMC9810477 36507649

[33] A. Cowie , J. Buckley , P. Doherty , et al. Standards and Core Components for Cardiovascular Disease Prevention and Rehabilitation. Published online April 1, 2019, 10.1136/heartjnl-2018-314206;. PMC6580752 30700518

