# Supplementary material for: Developing Glow: A Co‐Designed Support Framework Integrating Behavioural and Psychological Needs in Coeliac Disease
Source: Health Expect. 2026 Oct 8;29(5):e70905. doi: 10.1111/hex.70905 (PMC13647493; doi:10.1111/hex.70905)
Supplement: Supplementary file 1 [file HEX-29-e70905-s001.docx]

**Appendix 1. Research team positionality statement**

The research team brought a range of personal and professional identities that shaped how we engaged with participants, interpreted data, and co-constructed the emerging care framework. The team included individuals with lived experience of coeliac disease, a trainee health psychologist, an experienced psychologist, a psychologist with expertise in anxiety and coeliac disease, with team members from Global Majority backgrounds. All members of the team identified as female.

These positionalities influenced both the sensitivities we brought to the topic and the questions we asked. Lived experience created an insider awareness of the emotional and practical realities of gluten-free diet management, while professional training in psychology shaped our attentiveness to behaviour change processes, identity impacts, and psychological load. The presence of Global Majority researchers added perspectives on cultural, dietary, and healthcare inequities. Rather than seeking neutrality, we engaged in ongoing reflexive dialogue, through team debriefings, reflexive journaling, and explicit discussion of how our experiences and assumptions were shaping interpretation. Our aim was not to remove influence but to make it visible, examine it critically, and ensure that the developing framework remained grounded in the diverse realities shared by participants rather than the perspectives of the research team alone.
